# Computed Tomography-Based Radiomic Nomogram to Predict Occult Pleural Metastasis in Lung Cancer

**DOI:** 10.3390/curroncol32040223

**Published:** 2025-04-11

**Authors:** Xiaoyi Zhao, Heng Zhao, Kongxu Dai, Xiangyu Zeng, Yun Li, Feng Yang, Guanchao Jiang

**Affiliations:** 1 Department of Thoracic Surgery, Peking University People’s Hospital, No. 11 Xizhimen South Street, Xicheng District, Beijing 100044, China; 1410122808@pku.edu.cn (X.Z.); 13683139396@126.com (H.Z.); dkx980615@pku.edu.cn (K.D.); 231121334@pku.edu.cn (X.Z.); surgeonli@hotmail.com (Y.L.); 2 Thoracic Oncology Institute, Peking University People’s Hospital, Beijing 100044, China

**Keywords:** occult pleural metastasis, nomogram, CT-based radiomic, lung cancer

## Abstract

Objectives: The preoperative identification of occult pleural metastasis (OPM) in lung cancer remains a crucial clinical challenge. This study aimed to develop and validate a predictive model that integrates clinical information with chest CT radiomic features to preoperatively identify patients at risk of OPM. Methods: This study included 50 patients diagnosed with OPM during surgery as the positive training cohort and an equal number of nonmetastatic patients as the negative control cohort. Using least absolute shrinkage and selection operator (LASSO) logistic regression, we identified key radiomic features and calculated radiomic scores. A predictive nomogram was developed by combining clinical characteristics and radiomic scores, which was subsequently validated with data from an additional 545 patients across three medical centers. Results: Univariate and multivariate logistic regression analyses revealed that carcinoembryonic antigen (CEA), the neutrophil-to-lymphocyte ratio (NLR), the clinical T stage, and the tumor–pleural relationship were significant clinical predictors. The clinical model alone achieved an area under the curve (AUC) of 0.761. The optimal integrated model, which combined radiomic scores from the volume of interest (VOI) with the CEA and NLR, demonstrated an improved predictive performance, with AUCs of 0.890 in the training cohort and 0.855 in the validation cohort. Conclusions: Radiomic features derived from CT scans show significant promise in identifying patients with lung cancer at risk of OPM. The nomogram developed in this study, which integrates CEA, the NLR, and radiomic tumor area scores, enhances the precision of preoperative OPM prediction and provides a valuable tool for clinical decision-making.

## 1. Introduction

Lung cancer is a major contributor to cancer-related morbidity and mortality worldwide, with 2.5 million new cases and 1.8 million deaths recorded in 2022, accounting for 18.7% of all cancer-related fatalities [1]. Metastasis is the leading cause of mortality in lung cancer patients, with pleural metastasis (PM) being a common pattern of metastasis due to its nearby anatomical location, which typically presents on computed tomography (CT) scans as malignant effusions, pleural nodules, or irregular pleural thickening. Pleural metastasis in lung cancer is classified as M1a in the eighth edition of the IASLC TNM staging system, with a median survival time of only 11.5 months and a 10% 5-year overall survival rate [2].

Lung cancer with PM exhibits considerable heterogeneity and may elude detection because of the absence of typical clinical symptoms and imaging signs. Cases lacking direct symptoms related to pleural metastasis (e.g., chest pain), which are unexpectedly found during surgery and confirmed by a pathological biopsy, are referred to as occult pleural metastasis (OPM). The incidence of OPM remains uncertain, as indicated by varying rates from 0.9% to 5.26% in large-scale retrospective studies [3,4,5,6]. Notably, patients with occult PM tend to have a more favorable prognosis than those with clinically evident PM [7]. Hence, the timely identification of such patients prior to surgery is crucial to avoid needless surgical interventions and to determine the most appropriate treatment strategy.

The advent of radiomics represents a significant advancement in the extraction of intricate imaging data from CT scans with the potential to distinguish benign from malignant lung cancer, assess the efficacy of lung cancer treatments, and forecast outcomes [8,9,10]. Studies have indicated that the CT texture analysis of lung tumors is valuable for predicting occult lymph node metastasis in patients with lung cancer [11]. Our objective was to assess the predictive value of the CT radiomic features of lung cancer and the adjacent pleural areas in identifying OPM.

## 2. Materials and Methods

### 2.1. Patients

This retrospective study was endorsed by the ethics committee of Peking University People′s Hospital and was granted a waiver for informed consent. In this study, a case–control design (1:1 matching) was used to retrospectively collect data from lung cancer patients who underwent surgical treatment at our center from January 2015 to December 2022. Fifty patients with OPM of lung cancer who met the inclusion/exclusion criteria were selected as the positive group of the training cohort, and fifty patients were selected from the pool of patients without pleural metastasis of lung cancer at the same time as the negative control group. The age, sex, and BMI of the two groups of patients were matched. On the basis of the principle of continuous enrolment, 545 lung cancer patients were enrolled from our center and two external centers from January 2023 to June 2024 for model validation, including 25 patients positive for OPM of lung cancer and 520 patients without pleural metastasis.

**Figure 1:**
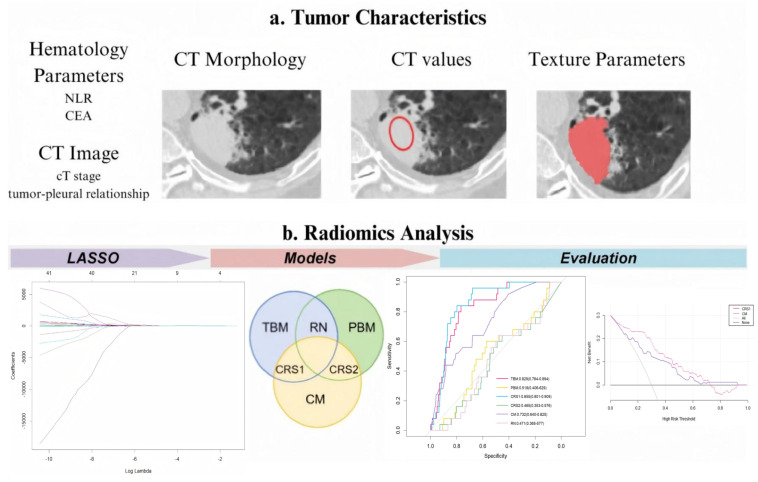
Experimental design flowchart. LASSO, least absolute shrinkage and selection operator; TBM, tumor-based model; PBM, pleura-based model; RN, radiomic nomogram; CM, clinical model; CRS1, combined radiomic signature 1; and CRS2, combined radiomic signature 2. Red hollow circles: Indicate regions where conventional CT parameters (e.g., CT stage, tumor-pleural relationship) were assessed. Red solid scribbles: Delineate volume of interest (VOI) for radiomics feature extraction.

The inclusion criteria were as follows: (1) the presence of a primary pulmonary lesion with a solid component ≥ 1 cm in maximum diameter on CT imaging, without features suggestive of pleural metastasis (e.g., multiple pleural nodules of pleural/pericardial effusion); (2) consecutive acquisition of chest CT and thoracoscopy at the same institution; and (3) histopathological confirmation of primary lung malignancy through surgical specimens. The exclusion criteria were as follows: (1) previous ipsilateral thoracic surgery; (2) history of recent pleurisy or malignant lesions; (3) interval > 2 weeks between CT scans and thoracoscopy; (4) CT artefacts affecting pleural lesion assessment; and (5) radiological evidence of distant metastases on preoperative evaluation. The overall framework is illustrated in Figure 1.

### 2.2. CT Image Acquisition

Participants underwent scanning with a Siemens SOMATOM Force CT scanner (Siemens Healthineers, Shanghai, China) at our facility and Philips 64-row CT (Philips Healthcare, Suzhou, China) at other sites. All patients were instructed to hold their breath while lying in the supine position to minimize potential motion artefacts induced by respiratory movements. A spiral scanning technique was employed with the following parameters: a tube voltage of 120 kVp, automatic tube current modulation, and a field of view covering the entire lung region.

For image reconstruction, an iterative reconstruction method was utilized. The display range of the Hounsfield unit (HU) was set to [−1024, 3071], which was divided into 164 discrete bins using a fixed bin width of 25 HU. This binning strategy was implemented to reduce image noise and enhance image quality. The reconstruction parameters included a slice thickness of 3 mm and a reconstruction interval of 1.5 mm, enabling overlapping reconstruction to improve spatial continuity. A high-resolution convolution kernel (e.g., Bv40 in Siemens systems) optimized for detailed soft tissue visualization was applied during the reconstruction process.

### 2.3. PM Status Determination

In this study, the status of pleural metastasis was determined using internationally accepted pathological diagnostic criteria. All enrolled patients underwent thoracoscopy with systematic exploration of the pleural surface by an experienced thoracic surgeon. Tissue samples (≥3 mm in diameter) were obtained using standardized biopsy forceps techniques for pleural lesions that were suspected by gross intraoperative observation (e.g., nodular thickening, abnormal congestion, or texture changes). After fixation with 4% neutral formaldehyde, the samples were subjected to histopathological analysis by senior pathologists, including infiltration of tumor cells into the subcutaneous connective tissue of the interpleura or the whole visceral pleura. The final diagnosis should be confirmed by two pathologists independently and unanimously as the objective basis for the presence or absence of pleural metastasis.

### 2.4. Data Collection and Analysis

**Figure 2:**
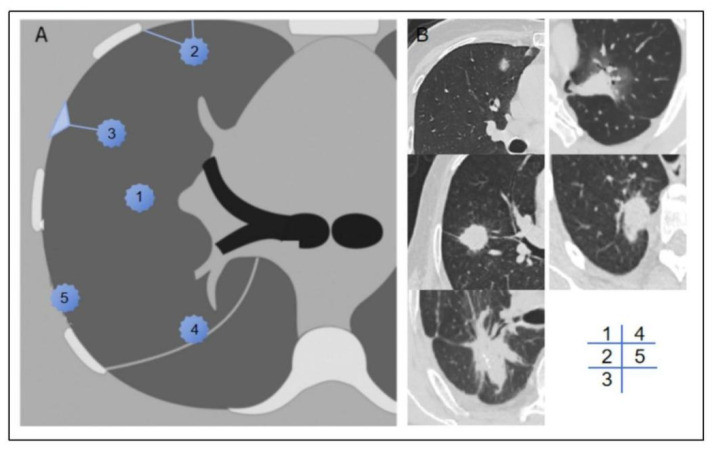
Schematic diagram (**A**) and representative imaging examples (**B**) of type 1–5 tumor–pleura relationships. In type 1, tumor is located inside lung and is unrelated to pleura. In type 2, tumor is not in contact with pleura, and one or more linear or striated pleural tags are visible. In type 3, tumor is not in contact with pleura, and one or more linear or striated pleural tags with soft tissue components at pleural end are visible. In type 4, tumor is in contact with interlobar pleura. In type 5, tumor is in contact with parietal pleura.

We documented the demographic information, laboratory test results, and tumor marker levels of all enrolled patients. These data included variables such as sex, age, BMI, smoking history, neutrophil-to-lymphocyte ratio (NLR), platelet-to-lymphocyte ratio (PLR), lymphocyte-to-monocyte ratio (LMR), prothrombin time (PT), activated partial thromboplastin time (APTT), fibrinogen (FIB), D-dimer levels, and a panel of tumor markers, including carcinoembryonic antigen (CEA), carbohydrate antigen 125 (CA125), carbohydrate antigen 19-9 (CA19-9), human CYFRA21-1 antigen (CYFRA21-1), and neuron-specific enolase (NSE). Preoperative CT scans were utilized to assess the patients’ clinical T stage (cT stage) and to evaluate the relationship between the tumor and pleura. Based on previous studies [12], we classified the tumor–pleura relationship into five distinct types, ranging from tumors confined to the lung parenchyma to those in direct contact with the parietal pleura (as illustrated in Figure 2).

Preoperative chest CT scans in DICOM format were collected, and high-density lesion contours were semiautomatically or manually delineated at the lesion edges using ITK-SNAP software (version 4.0.2). The term “volume of interest” (VOI) refers to the entire primary lesion observed on CT scans. First, a bounding box was drawn to provide spatial constraints for the algorithm, and the initial contour was generated on the basis of image features (grey gradient, texture similarity). The segmentation results were subsequently updated dynamically by adjusting the control points or correcting the boundaries, forming a closed loop of segmentation–feedback–resegmentation, and checking to ensure that the tumor lesions in each cross section from the top to the bottom of the nodule had been outlined completely. A manual segmentation method was used to delineate the pleural region of interest, called the ROI. The largest level of the tumor was selected on the CT cross section, and the nearest pleural point on the edge of the nodule on the level was taken as the starting point (avoiding the ribs). A 2 cm long pleural line was drawn along the intercostal direction and was extended to the upper and lower continuous levels. Finally, a pleural area of 2 cm × 2 cm was marked, and complete coverage was confirmed by 3D images. Two thoracic surgeons independently annotated the images, and a senior surgeon resolved any major discrepancies (Supplementary Figure S1). All annotations were performed using a window level of −500 HU and a window width of 1600 HU.

In accordance with PyRadiomics standards [13], we extracted 121 radiomic features from both the VOI and the ROI. These features were categorized into seven groups: first-order features, shape features, grey-level co-occurrence matrix (GLCM), grey-level size zone matrix (GLSZM), grey-level run-length matrix (GLRLM), neighboring grey tone difference matrix (NGTDM), and grey-level dependence matrix (GLDM). Univariate analysis, multivariate analysis, and least absolute shrinkage and selection operator (LASSO) regression were employed to select radiomic features from the VOI and ROI data. Additionally, we calculated the correlation coefficients between each pair of features to directly assess collinearity and redundancy, ensuring that highly correlated features (e.g., |correlation| > 0.7) were not included simultaneously in the final model. The resulting radiomic scores for the tumor area and pleural area were used as predictive factors in this study.

### 2.5. Nomogram Construction and Validation

Clinically significant variables (*p* < 0.05) identified through univariate analysis were further evaluated using multivariate logistic regression analysis. Nomogram prediction models, including clinical models, radiomic models, and combined models, were constructed on the basis of clinical features and/or radiomics scores. During model development, we used a single random split validation, dividing the data randomly into 30% for modelling and 70% for validation.

The predictive performance of each nomogram model was assessed using the area under the curve (AUC), specificity, sensitivity, and accuracy. The DeLong test was employed to determine the significance of the differences in the AUC values among the models. The Hosmer–Lemeshow (H-L) test was applied to evaluate the calibration of the nomogram models by comparing the predicted probabilities with the observed outcomes. In addition, we used bootstrap resampling to further evaluate model stability and uncertainty. Moreover, decision curve analysis (DCA) and a clinical impact curve (CIC) were performed to quantify the net benefits at varying threshold probabilities and to assess the clinical utility of the radiomic nomogram.

### 2.6. Statistical Analyses

Image delineation was performed using ITK-SNAP software (version 4.0.2), radiomic features were extracted using Python software (version 3.12), and all other statistical analyses were conducted using R software (version 4.3.3). A two-sided *p* value less than 0.05 was considered statistically significant.

## 3. Results

### 3.1. Clinical Characteristics

A total of 100 lung cancer patients were ultimately included in the modelling cohort, including 50 patients with OPM and 50 patients without OPM, and the cohorts were matched for age, sex, and BMI. The sex distribution of the enrolled patients was balanced (45 males), with an average age of 59.15 years and a median BMI of 24.53 kg/m^2^, which was consistent with the common characteristics of the Asian patients. The validation cohort included 545 patients from three medical centers and 25 patients positive for OPM, accounting for 4.59%, which was consistent with the incidence of OPM in the real world. To verify the generalizability of the prediction model, a comparative analysis of the baseline characteristics of the cohorts was performed, as shown in Table 1. The two cohorts demonstrated well-balanced distributions of 17 out of 19 clinical characteristics (*p* > 0.05), with the exception of the CEA level and the tumor–pleural spatial relationship. Notably, these two distinct variables were precisely incorporated into the predictive model, as the modelling cohort strategically increased the proportion of positive cases to enhance the model’s discriminative power for target events. This comparability of baseline characteristics confirms the validity of the intergroup comparisons.

Within the training cohort, there were no notable differences in clinical attributes, such as age, sex, smoking history, PLR, LMR, PT, APTT, FIB, D-dimer levels, CA199, CA125, CYFA21-1, NSE, and pathological subtypes, between the two groups (*p* > 0.05), as detailed in Table 2. However, four clinical variables—NLR, CEA, cT stage, and tumor–pleural relationship—demonstrated a statistical significance in the univariate analysis (with *p* values of 0.016, 0.002, 0.004, and 0.005, respectively). These four clinical characteristics in the multivariate logistic regression were also statistically significant (with *p* values of 0.043, 0.010, 0.010, and 0.037, respectively), as shown in Table 3. Given the significant differences, all four features were used in the construction of the clinical model.

### 3.2. Radiomic Signature Discovery

After the univariate logistic regression analysis, multivariate logistic regression analysis, and LASSO regression analysis, five radiomic features from the VOI were selected to calculate the radiomic score of the tumor area. These features included shape_Maximum2DDiameterSlice, firstorder_Skewness, glcm_Idn, glcm_InverseVariance, and glrlm_HighGrayLevelRunEmphasis. Similarly, eight features from the ROI were chosen to determine the pleural radiomic score, including shape_SurfaceArea, firstorder_Kurtosis, firstorder_Maximum, glcm_Correlation, glszm_ZoneEntropy, glszm_GrayLevelNonUniformityNormalized, glszm_GrayLevelVariance, and ngtdm_Strength. The correlation coefficients for each feature are presented in Table 4, and the LASSO screening plots for the VOI and ROI are presented in Supplementary Figure S2. The correlation matrix of the final selected features is shown in Supplementary Table S1.

### 3.3. Development and Validation of Nomogram

**Figure 3:**
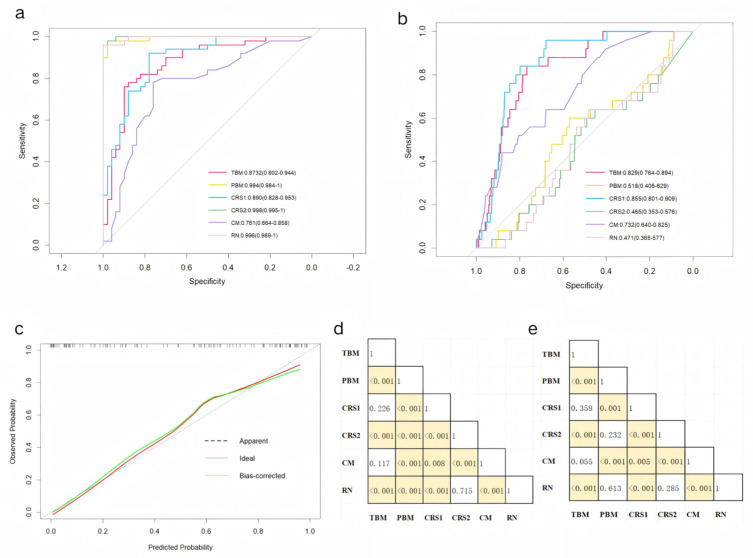
Receiver operating characteristic curves of the 6 predictive models in the (**a**) training and (**b**) validation cohorts. (**c**) Calibration curves for the radiomic nomogram in the validation cohort. DeLong test results between each pair of models in the (**d**) training and (**e**) validation cohorts. Yellow boxes represent *p* values < 0.05.

The pure clinical model (CM), which incorporates four statistically independent clinical predictors—the NLR, CEA level, cT stage, and tumor–pleural relationship—demonstrated the baseline predictive efficacy, with an AUC value of 0.761 in the training cohort and 0.732 in the validation cohort. Furthermore, three radiomic models were developed: the radiomic nomogram model (RN), which combines the VOI score and ROI score; the tumor-based model (TBM), which exclusively utilizes the VOI score; and the pleura-based model (PBM), which relies solely on the ROI score. Two composite models (CRS1: VOI + CEA + NLR; CRS2: ROI + CEA + NLR) further integrate the hematologic biomarkers. While an exceptional training performance was observed across all the models (RN: 0.996, TBM: 0.873, PBM: 0.994, CRS1: 0.890, and CRS2: 0.998), the validation outcomes revealed significant divergences (RN: 0.471, TBM: 0.829, PBM: 0.518, CRS1: 0.855, and CRS2: 0.465). Detailed accuracy metrics are catalogued in Table 5, while ROC curves, shown in Figure 3a,b, delineate the performance trajectory across cohorts.

The CRS1 exhibited a superior calibration concordance (Figure 3c). The DeLong test results, shown in Figure 3d,e, revealed differences in the AUCs among the various models in the training and validation cohorts, respectively. Combined with the data in Table 3, the AUC performance of the PBM, CRS2, and RN within the modelling cohort is clearly equally outstanding, markedly surpassing that of the other three models. In contrast, in the validation cohort, the PBM, CRS2, and RN all exhibited poor AUCs. Although the CRS1 and TBM exhibited comparable stability and discrimination, the CRS1 demonstrated superior clinical utility metrics in terms of accuracy, specificity, and sensitivity. The AUC of the CM is not as good as that of the CRS1 model, but the four features included in the CM were clinically obtained and can be used for the initial screening of patients.

**Figure 4:**
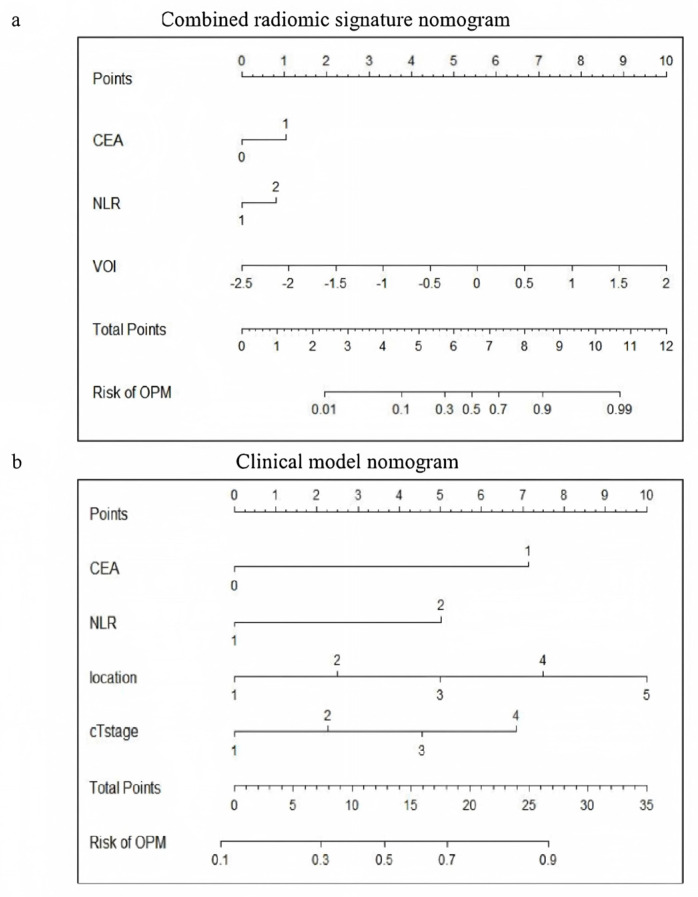
Visualization of (**a**) CRS1 nomogram and (**b**) CM nomogram. CRS1, combined radiomic signature 1; and CM, clinical model.

The visual nomogram of the CRS1 and CM is shown in Figure 4. For example, if a patient’s CEA is greater than normal, the NLR is greater than 2, the nodule is located in the interlobar pleura, and the clinical T stage is stage 3 then the corresponding scores in the CM are 7, 5, 7.4, and 4.6, and the total score is 24, corresponding to an OPM risk of close to 0.9.

### 3.4. Clinical Usage

**Figure 5:**
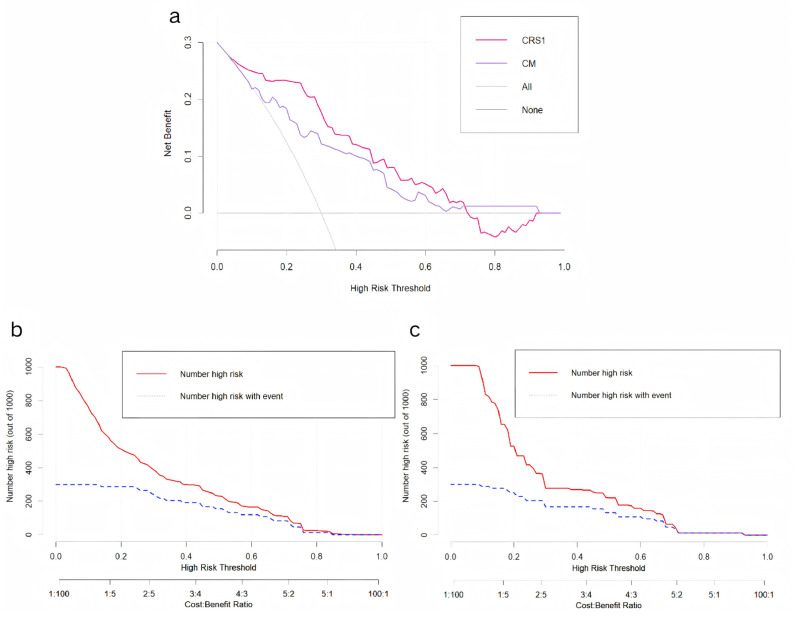
(**a**) Decision curve analysis of CRS1 model and CM. (**b**) Clinical impact curve of CRS1 model. (**c**) Clinical impact curve of CM.

A decision curve analysis (DCA) was conducted to evaluate the clinical utility of the nomogram, full thoracoscopy, and nonthoracoscopic surgical strategies (Figure 5a). Compared with the CM, full thoracoscopy, and nonthoracoscopic exploration protocols, the CRS1 model demonstrated superior clinical net benefits across most risk probability thresholds, indicating its increased value in guiding therapeutic decision-making.

A clinical impact curve (CIC) was generated to quantify the predictive performance of the models at varying threshold probabilities and to characterize the dynamic relationship between cost–benefit ratios and the identification of high-risk cohorts. The curves for both the CRS1 model and CM revealed a critical convergence pattern within the cost–benefit ratio range of 0.4–0.6, where the trajectories of the two curves exhibited parallel downwards trends (Figure 5b,c). This inflection point represents the equilibrium between the optimization of cost-effectiveness and the maximization of high-risk patient detection.

## 4. Discussion

To our knowledge, this study represents a pioneering effort in developing predictive models for OPM by integrating clinical and radiomic data. The combined CRS1 model, which incorporates CEA, the NLR, and the VOI, demonstrated a robust predictive accuracy and calibration in preoperatively identifying the presence or absence of OPM. The CM is recommended for initially screening patients in clinical practice, with the subsequent acquisition of radiomic features for high-risk individuals (identified by an elevated OPM probability) to undergo CRS1 model verification.

The anatomical proximity of the pleura to the lung tissue facilitates the direct infiltration of lung cancer cells into the adjacent pleural area. However, during the early stages of pleural metastasis, the absence of pleural thickening or effusion often complicates the detection via conventional CT imaging. Clinical observations indicate that even small tumors without lymph node metastasis may harbor occult pleural metastasis, highlighting the critical need for improved diagnostic accuracy to ensure precise staging and treatment planning. PET–CT imaging, which relies on detecting an abnormally elevated glucose metabolism in tumor cells, has significant limitations in identifying subcentimeter metastatic lesions (diameter <7–10 mm) because partial volume effects reduce standardized uptake values (SUVmax) below diagnostic thresholds (typically 2.5), resulting in metabolic signals indistinguishable from the background tissue. This contributes to false-negative rates of 22.9–41%, particularly for pulmonary micrometastases [3,14]. Furthermore, while thoracentesis and pleural biopsy provide a histopathological confirmation, their variable sensitivity (40–87%) and procedural invasiveness with associated complication risks limit their clinical utility. These limitations underscore the rationale for developing a CT-based radiomic model for preoperative OPM prediction, which noninvasively quantifies tumor heterogeneity patterns to a greater extent than metabolic or morphological assessments alone [7,15,16].

Previous studies have underscored the importance of hematological indicators, such as the NLR, PLR, LMR, PT, APTT, FIB, and D-dimer, in predicting occult peritoneal metastasis in patients with gastric cancer [17]. In addition, an elevated preoperative NLR was a strong independent predictor of extensive peritoneal metastasis in advanced gastric cancer patients [18,19]. Although the role of the NLR in the development of malignant tumors has not been clearly defined, studies have shown that a relatively high number of neutrophils can promote tumor growth, invasion, and metastasis by secreting the vascular endothelial growth factor (VEGF) and carcinogen M [20]. Neutrophils may also lead to cancer by releasing a high number of reactive oxidative substances [21]. Since CD4+ and CD8+ T cells provide tumor cell immunity, reduced lymphocyte numbers usually indicate the suppression of tumor immunity, providing favorable conditions for tumor progression [22]. Tumor progression is particularly rapid under the synergistic action of neutrophils and lymphocytes.

In the context of lung cancer, Li et al. identified an age under 50 years, elevated CEA, advanced *n* stage, adenocarcinoma histology, and pleural invasion as independent risk factors for OPM, with an AUC of 0.756 when these clinical factors were integrated [5]. The results of our study are essentially consistent with those of the above studies, especially in confirming the value of elevated CEA in the prediction of metastasis, whereas other tumor markers, such as CA125, CA19-9, CYFRA21-1, and NSE, have no significant value in the prediction of the occult pleural metastasis of lung cancer. A possible explanation for this finding is that CEA can promote the pleural invasion and implantation metastasis of tumor cells by activating the PI3K/AKT signaling pathway, increasing the expression and activity of mechanical metalloproteinases, and mediating the degradation of the extracellular matrix and basement membrane [23]. Additionally, the clinical *n* stage, which may be influenced by age-related factors or prior infections, was excluded from our predictive model because of its discordance with the pathological stage; there were no significant differences in the pathological subtype between the positive and negative groups in the modelling cohort. Furthermore, since our model is intended for the preoperative prediction of OPM and pathological information is not routinely available preoperatively in clinical practice, pathological subtypes were not included as predictive factors in our model.

Zhang et al. [12] categorized the tumor–pleura relationship into four types to predict occult lymphatic metastasis in patients with clinical stage IA lung adenocarcinoma. In our study, we further refined this classification by distinguishing between tumors in contact with the visceral pleura and those in contact with the parietal pleura. The results presented in this paper verify that our distinction between types 4 and 5 is statistically significant. The CM nomogram of the primary screening model also revealed that the risk of metastasis in type 5 patients was significantly greater than that in type 4 patients, and the difference reflected a total score increase of 10 points in the CM nomogram (Figure 4b). With the increasing classification grade (type 1 to type 5), the tumor gradually moves closer to the chest wall from the anatomical position, and there are more opportunities for implantation and metastasis. Type 5 lesions are most likely to implant into the parietal pleural surface. The density of lymphatic vessels in the parietal pleura is greater than that in the visceral pleura, which provides channels for cancer cell dissemination. Moreover, the interlobar pleura and costal pleura have different lymphatic drainage directions: the former is more likely to metastasize to hilar or mediastinal lymph nodes, whereas the latter is directly connected to the intercostal lymph node chain, forming the anatomical basis for implant metastasis [24]. In addition, pressure changes in the thoracic cavity and the relaxation and contraction of the lungs during respiratory movements may promote the shedding of cancer cells from the affected pleural site. Compared with type 4 lesions located in the interlobar fissure, type 5 lesions close to the intercostal space have a greater risk of shedding during respiratory movement; thus, the risk of metastasis is greater.

The radiomics features included in the VOI score, such as “shape_Maximum2DDiameterSlice”, “GLCM-Idn”, “GLCM-InverseVariance”, and “glrlm_HighGrayLevelRunEmphasis”, provide valuable insights into tumor characteristics. For example, the “shape_Maximum2DDiameterSlice” reflects tumor size and is closely associated with the clinical T stage. A larger maximum diameter is associated with a more advanced tumor stage and a greater likelihood of metastasis. The features “GLCM-Idn” and “GLCM-InverseVariance” measure tumor homogeneity, which may be correlated with malignancy and metastatic potential. Similarly, “glrlm_HighGrayLevelRunEmphasis” captures high-intensity regions within the tumor, potentially indicative of aggressive tumor behavior. Therefore, these features collectively contribute to the predictive power of the VOI radiomic score.

Among the six models constructed, three incorporating ROI models exhibited marked performance differences between the training and validation cohorts. The main reasons for the overtraining of the PBM, CRS2 model, and RN are as follows: First, the ROI delineation is challenging, especially in terms of precisely defining the outer edge of the parietal pleura. Different physicians’ delineation biases can lead to systematic biases in radiomic feature extraction. In contrast, the VOI delineation, which is based on semiautomatic tumor segmentation with manual correction, has better reproducibility. Second, the ROI texture features are significantly phase dependent on respiratory motion. In this retrospective study, although patients were given routine respiratory guidance during the CT image acquisition, it was difficult to ensure the proper respiratory cooperation in all cases. Third, due to the difficulty of manual delineation, completely delineating all the pleural areas on the lesion side is challenging. This study focused on ROIs in the rib pleura nearest the lesion. However, tumor cells might deposit in the lower part of the pleural cavity due to negative pressure and respiratory shear forces, potentially causing hidden metastases in distant rib pleural areas, even if none are found in the delineated region. This reduces the model’s sensitivity to the OPM status and increases the false-negative rate. Finally, errors in the ROI delineation are amplified by the sparsity-inducing properties of LASSO regression. This gives pseudo-correlated imaging features nonzero coefficients and eliminates true signal features due to collinearity, inverting the feature importance order.

In setting the high-risk thresholds for the CM and CRS1 model, we used decision curve analysis and clinical impact curves. For the CM, we set the threshold between 0.1 and 0.4, favoring a lower threshold to avoid missing high-risk patients, making it a good initial screening tool. For the CRS1 model, we chose a higher threshold of 0.3 to 0.6 to minimize false-positives, making it a good confirmatory tool. Specifically, patients with a CM score above 0.3 will undergo further radiomic feature extraction and verification using the CRS1 model. If the CRS1 score exceeds 0.6, it indicates a high risk of OPM. This CM initial screening → CRS1 model confirmation system enables effective patient risk stratification. Clinically, it allows doctors to use model-predicted values to inform patients of their OPM risk and discuss treatment options thoroughly, promoting shared decision-making. For high-risk OPM patients who are not ideal surgical candidates (due to poor general condition, low surgical tolerance, or unwillingness to undergo surgery), less invasive diagnostic methods, such as pleural lavage cytology or medical thoracoscopy, can be used to confirm pleural metastasis and guide treatment planning. This approach helps avoid unnecessary invasive procedures (such as thoracoscopy) that could delay comprehensive treatment. Conversely, for patients with good general health, high surgical tolerance, and a strong willingness to undergo surgery, preparations such as hyperthermic thoracic perfusion equipment and chemotherapeutic drugs (e.g., cisplatin) can be made in advance. During surgery, doctors should meticulously check for occult pleural metastases, focusing on areas such as the diaphragm dome and interlobar fissures, using methylene blue staining if needed, and perform frozen section analysis on suspicious lesions. Therefore, our prediction model is important for optimizing staging and treatment decisions in lung cancer patients.

This study has several limitations. First, its retrospective design may introduce inherent biases and confounding factors. Second, the low detection rate of OPM (4.29%) in our study resulted in an imbalance between positive and negative cases, which may affect the stability of the model and result in a high variability in the results. Additionally, this study lacks external validation using an independent dataset from another institution, because more than 70% of patients were from the primary institution. This approach limits the feasibility of standalone external validation. Future studies should validate these findings in larger, multicenter cohorts to ensure generalizability, and genomic information should be collected to enhance the causal interpretability of radiomic features. Third, the complex nature of the pleural region makes accurate delineation difficult, which can introduce segmentation errors. These errors may bias radiomic feature extraction, affecting the model performance. Variations in CT parameters across different institutions can further complicate the segmentation process and introduce variability in the extracted features. To address these challenges, improved imaging techniques and automated segmentation methods are needed. These advancements could increase the accuracy and consistency of pleural radiomic features, potentially improving the reliability of predictive models. Future research should explore the impact of segmentation errors and CT parameter differences on model outcomes and develop strategies to mitigate these effects.

## 5. Conclusions

In conclusion, our study demonstrates the potential of integrating clinical and radiomic data to predict OPM in lung cancer patients. The CRS1 model, in particular, offers a robust and practical tool for preoperative risk assessment, with the potential to improve clinical outcomes by guiding personalized treatment strategies. Future research should focus on refining radiomic features, validating models in diverse populations, and exploring the integration of additional biomarkers to further enhance predictive accuracy.

## Figures and Tables

**Table 1:** Clinical, radiological, and pathological characteristics.

Characteristic	Training Cohort (*n* = 100)	Validation Cohort (*n* = 545)	Statistic	*p*
Age (years)	59.15 ± 9.72	60.38 ± 7.79	t = −1.199	0.233
Sex			χ^2^ = 0.589	0.443
Male	45 (45.0)	268 (49.2)		
Female	55 (55.0)	277 (50.8)		
BMI, kg/m^2^	24.53 ± 3.35	24.31 ± 3.16	t = 0.646	0.519
Somke			χ^2^ = 0.686	0.408
Negative	69 (69.0)	398 (73.0)		
Positive	31 (31.0)	147 (27.0)		
NLR			χ^2^ = 1.761	0.185
<2	46 (46.0)	290 (53.2)		
≥2	54 (54.0)	255 (46.8)		
LMR			χ^2^ = 3.169	0.075
<5	86 (86.0)	426 (78.2)		
≥5	14 (49.0)	119 (21.8)		
PLR			χ^2^ = 0.469	0.493
<165	80 (80.0)	419 (76.9)		
≥165	20 (20.0)	126 (23.1)		
PT			χ^2^ = 0.039	0.843
<12 s	81 (81.0)	446 (81.8)		
≥12 s	19 (19.0)	99 (18.2)		
APTT			χ^2^ = 2.857	0.091
<28 s	75 (75.0)	448 (82.2)		
≥28 s	25 (25.0)	97 (17.8)		
FIB			χ^2^ = 2.757	0.097
<3 g/L	56 (56.0)	256 (47.0)		
≥3 g/L	44 (44.0)	289 (53.0)		
D-dimer			χ^2^ = 0.928	0.335
<0.5 mg/L	89 (89.0)	501 (91.9)		
≥0.5 mg/L	11 (11.0)	44 (8.1)		
CEA			χ^2^ = 26.702	<0.001 *
Normal	61 (61.0)	455 (83.5)		
Elevated	39 (39.0)	90 (16.5)		
CA199			χ^2^ = 1.065	0.302
Normal	89 (89.0)	502 (92.1)		
Elevated	11 (11.0)	43 (7.9)		
CA125			χ^2^ = 0.460	0.498
Normal	96 (96.0)	530 (97.3)		
Elevated	4 (4.0)	15 (2.7)		
NSE			χ^2^ = 3.272	0.070
Normal	96 (96.0)	493 (90.5)		
Elevated	4 (4.0)	52 (9.5)		
CYFRA21-1			χ^2^ = 2.035	0.154
Normal	96 (96.0)	501 (91.9)		
Elevated	4 (4.0)	44 (8.1)		
Pathology			χ^2^ = 0.892	0.827
Adenocarcinoma	93 (93.0)	505 (92.7)		
SCC	5 (5.0)	28 (5.1)		
Other NSCLC	2 (2.0)	8 (1.5)		
SCLC	0 (0.0)	4 (0.7)		
cT stage			χ^2^ = 5.098	0.165
T1	63 (63.0)	390 (71.6)		
T2	27 (27.0)	128 (23.5)		
T3	8 (8.0)	21 (3.9)		
T4	2 (2.0)	6 (1.1)		
Location			χ^2^ = 29.077	<0.001 *
Type 1	8 (8.0)	125 (22.9)		
Type 2	19 (19.0)	69 (12.7)		
Type 3	21 (21.0)	174 (31.9)		
Type 4	25 (25.0)	59 (10.8)		
Type 5	27 (27.0)	118 (21.7)		

The data are presented as the *n* (%) and mean ± standard deviation. The * after the *p*-value indicates that the feature is statistically significant. BMI: body mass index; LMR: lymphocyte-to-monocyte ratio; NLR: neutrophil-to-lymphocyte ratio; PLR: platelet-to-lymphocyte ratio; CEA: carcinoembryonic antigen; PT: prothrombin time; APTT: activated partial thromboplastin time; FIB: fibrinogen; CEA: carcinoembryonic antigen; CA199: carbohydrate antigen 19-9; CA125: carbohydrate antigen 125; CYFRA21-1: human CYFRA21-1 antigen; and NSE: neuron-specific enolase/air space.

**Table 2:** Univariate analysis of clinical features associated with OPM status in training cohort.

Characteristic	OPM− (*n* = 50)	OPM+ (*n* = 50)	Statistic	*p*
Age (years)	58.58 ± 8.31	59.72 ± 11.00	t = −0.585	0.560
Sex			χ^2^ = 1.010	0.315
Male	25 (50.0)	20 (40.0)		
Female	25 (50.0)	30 (60.0)		
BMI, kg/m^2^	24.77 ± 3.69	24.30 ± 3.00	t = 0.689	0.493
Somke			χ^2^ = 2.291	0.130
Negative	31 (62.0)	38 (76.0)		
Positive	19 (31.0)	12 (24.0)		
NLR			χ^2^ = 5.797	0.016 *
<2	29 (58.0)	17 (34.0)		
≥2	21 (42.0)	33 (66.0)		
LMR			χ^2^ = 332	0.564
<5	42 (84.0)	44 (88.0)		
≥5	8 (16.0)	6 (12.0)		
PLR			χ^2^ = 2.250	0.134
<165	43 (86.0)	37 (74.0)		
≥165	7 (14.0)	13 (26.0)		
PT			χ^2^ = 0.065	0.799
<12 s	40 (80.0)	41 (82.0)		
≥12 s	10 (20.0)	9 (18.0)		
APTT			χ^2^ = 1.333	0.248
<28 s	40 (80.0)	35 (70.0)		
≥28 s	10 (20.0)	15 (30.)		
FIB			χ^2^ = 0.649	0.420
<3 g/L	30 (60.0)	26 (52.0)		
≥3 g/L	20 (40.0)	24 (48.0)		
D-dimer			χ^2^ = 0.919	0.338
<0.5 mg/L	46 (92.0)	43 (86.0)		
≥0.5 mg/L	4 (8.0)	7 (14.0)		
CEA			χ^2^ = 9.458	0.002 *
Normal	38 (76.0)	23 (46.0)		
Elevated	12 (24.0)	27 (54.0)		
CA199			χ^2^ = 2.554	0.110
Normal	47 (94.0)	42 (84.0)		
Elevated	3 (6.0)	8 (16.0)		
CA125			χ^2^ = 1.042	0.307
Normal	47 (94.0)	49 (98.0)		
Elevated	3 (6.0)	1 (2.0)		
NSE			χ^2^ = 0.000	1.000
Normal	48 (96.0)	48 (96.0)		
Elevated	2 (4.0)	2 (4.0)		
CYFRA21-1			χ^2^ = 1.042	0.307
Normal	49 (98.0)	47 (94.0)		
Elevated	1 (2.0)	3 (6.0)		
Pathology			χ^2^ = 0.211	0.900
Adenocarcinoma	46 (92.0)	47 (94.0)		
SCC	3 (6.0)	2 (4.0)		
Other NSCLC	1 (2.0)	1 (2.0)		
SCLC	0 (0.0)	0 (0.0)		
cT stage			χ^2^ = 13.069	0.004 *
1	40 (63.0)	23 (46.0)		
2	8 (16.0)	18 (38.0)		
3	2 (4.0)	6 (12.0)		
4	0 (0.0)	2 (4.0)		
Location			χ^2^ = 14.782	0.005 *
Type 1	5 (10.0)	3 (6.0)		
Type 2	14 (28.0)	5 (10.0)		
Type 3	14 (28.0)	7 (14.0)		
Type 4	6 (12.0)	19 (38.0)		
Type 5	11 (22.0)	16 (32.0)		

The * after the *p*-value indicates that the feature is statistically significant. OPM+: with occult pleural metastasis; OPM−: without occult pleural metastasis; LMR: lymphocyte-to-monocyte ratio; NLR: neutrophil-to-lymphocyte ratio; PLR: platelet-to-lymphocyte ratio; CEA: carcinoembryonic antigen; PT: prothrombin time; APTT: activated partial thromboplastin time; FIB: fibrinogen; CEA: carcinoma embryonic antigen; CA199: carbohydrate antigen 19-9; CA125: carbohydrate antigen 125; CYFRA21-1:human CYFRA21-1 antigen; NSE: neuron-specific enolase; and location: tumor–pleural relationship.

**Table 3:** Multivariate analysis of clinical features associated with OPM status in training cohort.

Feature	β	Std. Error	Z Value	Wald χ^2^	Pr (|Z|)	OR	95%CI
NLR	0.904	0.446	2.027	4.107	0.043	2.470	1.030–5.921
CEA	1.297	0.501	2.589	6.705	0.010	3.657	1.371–9.760
cT stage	0.973	0.379	2.567	6.590	0.010	2.645	1.259–5.557
Location	0.386	0.185	2.085	4.348	0.037	1.471	1.023–2.113

NLR: neutrophil-to-lymphocyte ratio; CEA: carcinoembryonic antigen; and location: tumor–pleural relationship.

**Table 4:** Correlation coefficients of radiomic features.

Variable of VOI	Coef
(Intercept)	−16.93664
shape_Maximum2DDiameterSlice	0.000979729
firstorder_Skewness	−0.2107765
glcm_Idn	16.18972
glcm_InverseVariance	2.98917
glrlm_HighGrayLevelRunEmphasis	0.000280008
Variable of ROI	Coef
(Intercept)	−23.359305349
shape_SurfaceArea	0.001080420
firstorder_Kurtosis	1.197099060
firstorder_Maximum	0.003960438
glcm_Correlation	10.638743549
glszm_GrayLevelNonUniformityNormalized	−1.595754622
glszm_GrayLevelVariance	0.049673685
glszm_ZoneEntropy	0.480000350
ngtdm_Strength	0.782355538

**Table 5:** Performance of different predictive models.

Models	Training Cohort				Validation Cohort			
	AUC (95%Cl)	ACC	SPE	SEN	AUC (95%Cl)	ACC	SPE	SEN
RN(VOI + ROI)	0.996(0.989–1)	0.988	0.998	0.978	0.471(0.365–0.577)	0.490	0.483	0.640
CRS1(CEA + NLR + VOI)	0.890(0.828–0.953)	0.850	0.780	0.920	0.855(0.801–0.909)	0.770	0.767	0.840
CRS2(CEA + NLR + ROI)	0.998(0.995–1)	0.980	0.980	0.980	0.465(0.353–0.576)	0.460	0.452	0.640
TBM(VOI)	0.873(0.802–0.944)	0.830	0.900	0.760	0.829(0.764–0.894)	0.691	0.679	0.960
PBM(ROI)	0.994(0.984–1)	0.980	0.980	0.980	0.518(0.406–0.629)	0.521	0.517	0.600
CM(CEA + NLR + cTstage + T–P relationship)	0.761(0.644–0.858)	0.770	0.760	0.780	0.732(0.640–0.825)	0.793	0.806	0.520

AUC, area under the curve; Cl, confidence interval; ACC, accuracy; SPE, specificity; and SEN, sensitivity.

## Data Availability

The datasets used and analyzed during the current study are available from the corresponding author upon reasonable request.

## Supplementary Materials

The following supporting information can be downloaded at https://www.mdpi.com/article/10.3390/curroncol32040223/s1: Supplementary Figure S1. An example image is shown using ITK-SNAP software to outline the VOI (red) and ROI (yellow) with coronal CT image on the left and 3D image on the right; Supplementary Figure S2. LASSO logistic regression of features from the primary tumor (A, B) and pleura (C, D). LASSO regression can reduce the feature dimension by shrinking the coefficients of some features to zero and further selecting key features. Supplementary Table S1. The correlation matrix of final selected features in VOI and ROI.
