# Supplementary material for: Computed Tomography-Based Radiomic Nomogram to Predict Occult Pleural Metastasis in Lung Cancer
Source: Curr Oncol. 2025 Apr 11;32(4):223. doi: 10.3390/curroncol32040223 (PMC12025487; doi:10.3390/curroncol32040223)
Supplement: Supplementary file 1 [file curroncol-32-00223-s001.zip › curroncol-3491864-Table S1.pdf]

Supplementary Table S1 The correlation matrix of final selected features in VOI and ROI.

Features in VOI	shape_Maximum 2DDiameterSlice	firstorder_ Skewness	glcm_Idn	glcm_Inverse Variance	glrlm_HighGray LevelRunEmphasis
shape_Maximum 2DDiameterSlice	1.000	-0.590	0.540	0.408	0.587
firstorder_ Skewness	-0.590	1.000	-0.680	-0.659	-0.650
glcm_Idn	0.540	-0.680	1.000	0.670	0.506
glcm_Inverse Variance	0.408	-0.659	0.670	1.000	0.322
glrlm_HighGray LevelRunEmphasis	0.587	-0.650	0.506	0.322	1.000

Features in ROI	shape_Surface Area	firstorder_ Kurtosis	firstorder _Maximum	glcm_ Correlation	glcm_MCC	glszm_GrayLevelNon UniformityNormalized	glszm_Gray LevelVariance	glszm_ ZoneEntropy	ngtdm_ Strength
shape_SurfaceArea	1.000	0.267	0.428	0.345	0.226	-0.298	0.251	0.567	-0.070
firstorder_Kurtosis	0.267	1.000	0.260	0.210	0.232	-0.049	0.141	0.239	0.450
firstorder_Maximum	0.428	0.260	1.000	0.246	0.354	-0.577	0.572	0.607	0.476
glcm_Correlation	0.345	0.210	0.246	1.000	0.676	-0.442	0.421	0.636	0.325
glcm_MCC	0.226	0.232	0.354	0.676	1.000	-0.414	0.357	0.397	0.478
glszm_GrayLevelNonUniformityNormalized	-0.298	-0.049	-0.577	-0.442	-0.414	1.000	-0.615	-0.652	-0.653
glszm_GrayLevel Variance	0.251	0.141	0.572	0.421	0.357	-0.615	1.000	0.687	0.690
glszm_ZoneEntropy	0.567	0.239	0.607	0.636	0.397	-0.652	0.687	1.000	0.490
ngtdm_Strength	-0.070	0.450	0.476	0.325	0.478	-0.653	0.690	0.490	1.000
