# Supplementary figures and images for: Computed Tomography-Based Radiomic Nomogram to Predict Occult Pleural Metastasis in Lung Cancer
Source: Curr Oncol. 2025 Apr 11;32(4):223. doi: 10.3390/curroncol32040223 (PMC12025487; doi:10.3390/curroncol32040223)

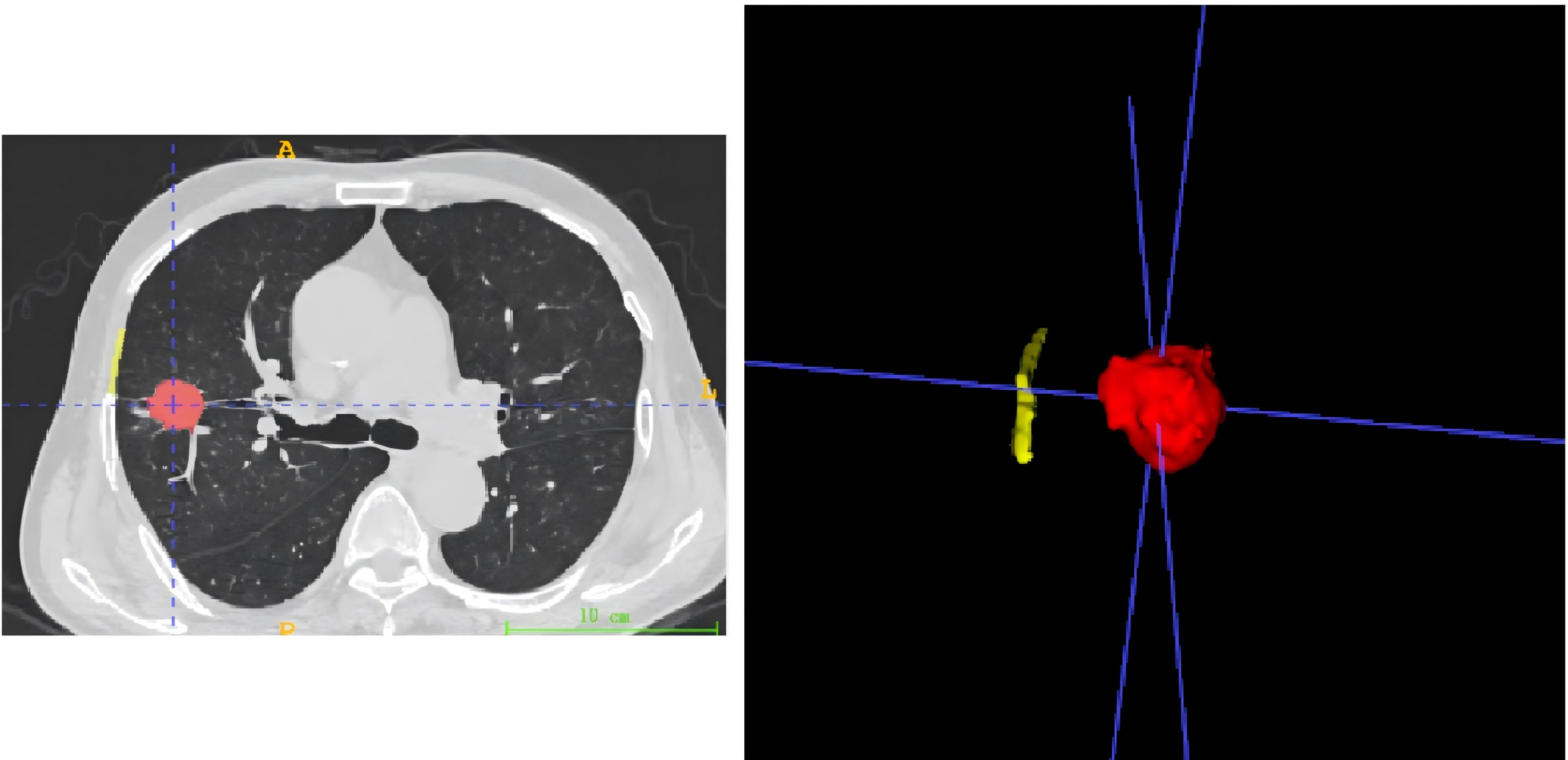

Supplement: Supplementary file 1 [file curroncol-32-00223-s001.zip › Figure S1.jpg]

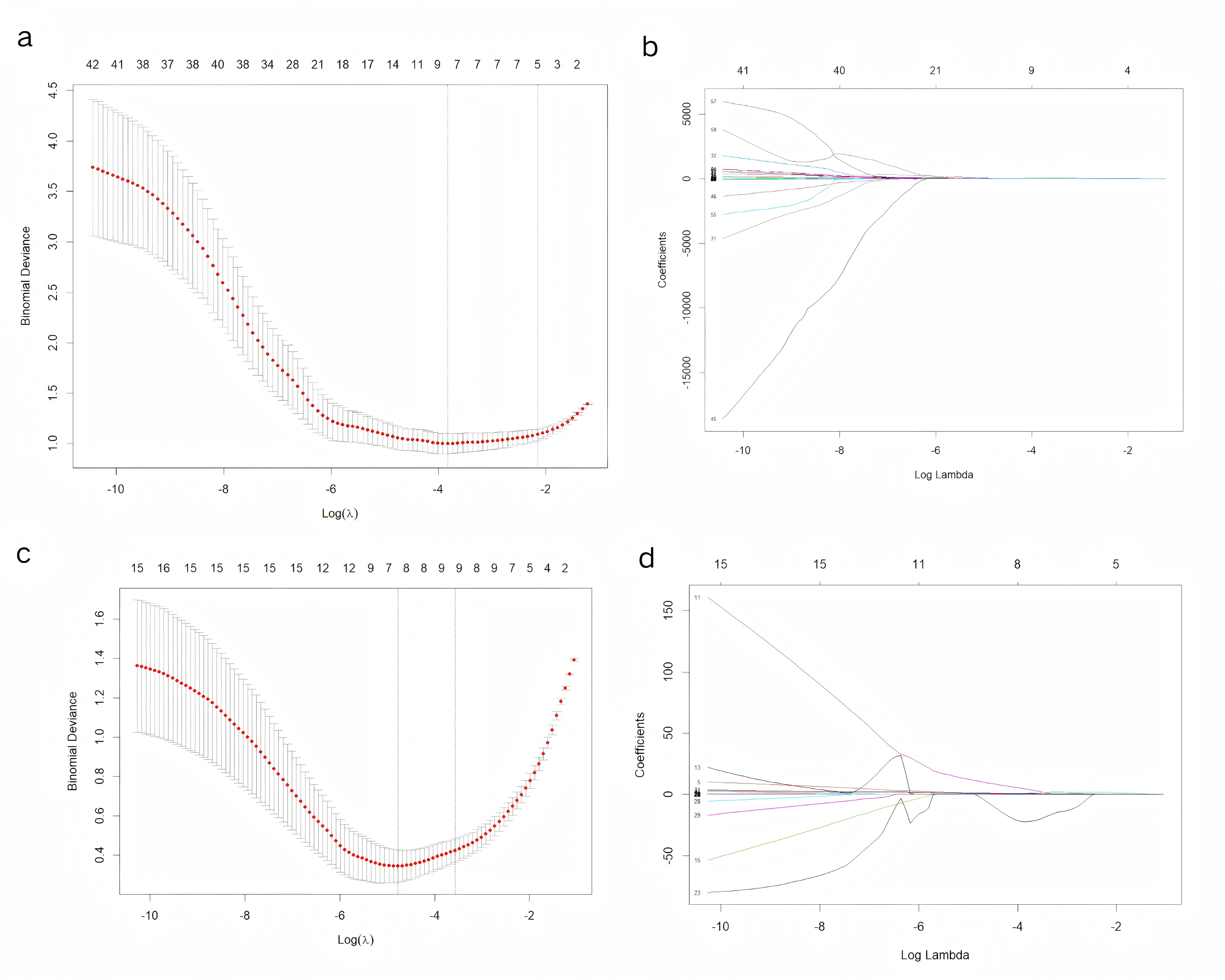

Supplement: Supplementary file 1 [file curroncol-32-00223-s001.zip › Figure S2.png]
